# Piceatannol Induces Mitochondrial Dysfunction in *Toxoplasma gondii*

**DOI:** 10.3390/microorganisms13061203

**Published:** 2025-05-25

**Authors:** Zhenhe Liu, Haolong Qiu, Yucong Jiang, Yuxi Mo, Linlin Lu, Yan Wang, Dandan Hu, Xingju Song

**Affiliations:** 1 Guangxi Key Laboratory of Animal Breeding, Disease Control and Prevention, College of Animal Science and Technology, Guangxi University, Nanning 530004, China; 13768115231@163.com (Z.L.); q961616555@163.com (H.Q.); jiangyucong0401@163.com (Y.J.); 13873563575@163.com (Y.M.); 18172072674@163.com (L.L.); 18275863237@163.com (Y.W.); 2 Shizhong District Animal Disease Prevention and Control Center, Leshan 614000, China; 3 Guangxi Zhuang Autonomous Region Engineering Research Center of Veterinary Biologics, Nanning 530004, China

**Keywords:** *Toxoplasma gondii*, piceatannol, mitochondria, autophagy, RNA-seq

## Abstract

*Toxoplasma gondii*, an obligate intracellular protozoan parasite infecting nucleated cells of warm-blooded vertebrates, causes severe complications in immunocompromised hosts. Current therapies remain limited by suboptimal efficacy and toxicity, necessitating novel anti-toxoplasmic agents. Piceatannol (PIC), a natural stilbenoid, demonstrates multifaceted bioactivity including antimicrobial and anti-parasitic effects, suggesting therapeutic potential against *T. gondii*. Our previous study revealed PIC’s potent anti-parasitic activity, selectively inhibiting *T. gondii* proliferation and altering parasite morphology without host cytotoxicity. In this study, mechanistic analyses indicated that PIC disrupts mitochondrial integrity in tachyzoites, reducing mitochondrial membrane potential and ATP production while elevating ROS levels. Transcriptomic profiling identified significant suppression of oxidative phosphorylation-related genes, consistent with mitochondrial dysfunction. These findings establish PIC as a promising candidate targeting *T. gondii* through the mechanism of mitochondrial impairment.

## 1. Introduction

*Toxoplasma gondii*, an obligate intracellular apicomplexan parasite, represents a major zoonotic pathogen affecting humans and homeothermic vertebrates worldwide. While establishing typically asymptomatic chronic infections in immunocompetent hosts, this parasite poses life-threatening risks to immunocompromised populations including patients with HIV/AIDS, transplant recipients, and individuals undergoing chemotherapy, frequently manifesting as toxoplasmic encephalitis, pneumonitis, or chorioretinitis [1,2,3]. Vertical transmission during pregnancy may induce congenital toxoplasmosis, associated with severe neurological sequelae, spontaneous abortion, and stillbirth [4,5]. The current therapeutic method combining sulfadiazine and pyrimethamine demonstrates limited efficacy against chronic infections and carries substantial toxicity risks, including hematologic suppression and teratogenic effects [6,7,8]. Alternative regimens employing spiramycin, azithromycin, or atovaquone show reduced anti-parasitic potency compared to conventional therapy [9,10,11], underscoring the critical need for novel therapeutic agents.

Phytochemicals have gained prominence as reservoirs of anti-toxoplasmic compounds [12], with multiple plant-derived substances exhibiting parasite inhibition. Artemisinin analogs from *Artemisia annua*, camphor derivatives from *Cinnamomum camphora*, and bioactive constituents from *Lippia multiflora* and *Vernonia colorata* demonstrate dose-dependent suppression of *T. gondii* proliferation [13]. Essential oils from *Lavandula angustifolia*, *Geranium longifolium*, and *Eurycoma longifolia* (TAF355/TAF401), along with ginkgolic acid from *Ginkgo biloba*, exhibit stage-specific anti-parasitic activity through undefined mechanisms [14,15,16,17]. Among these, the stilbenoid piceatannol (PIC), a hydroxylated resveratrol analog ubiquitously present in *Passiflora edulis*, *Vaccinium* spp., and *Vitis vinifera*, displays broad-spectrum bioactivity including antiproliferative, immunomodulatory, and antimicrobial effects [18,19]. Notably, PIC demonstrates potent protozoacidal activity against *T. gondii*, *Leishmania donovani*, and *Acanthamoeba castellanii* via undefined molecular targets [20].

Our recent investigations revealed PIC’s dose-dependent anti-tachyzoite activity (EC50 = 28.10 μM) with preserved host-cell viability at therapeutic concentrations [21]. Phenotypic analysis demonstrated PIC-induced replication anomalies in ~60% of treated tachyzoites, including aberrant endodyogeny patterns, with tripartite tachyzoites being found in a single parasitic vacuole (PV). While preliminary evidence in bacteria suggested mitochondrial ATP synthase as a potential molecular target [22], the precise mechanism underlying PIC’s anti-*Toxoplasma* effects remains uncharacterized. To address this knowledge gap, we conducted immunofluorescent and functional analyses of PIC-treated tachyzoites, revealing mitochondrial cristae disorganization accompanied by membrane potential collapse, ATP depletion, and reactive oxygen species (ROS) accumulation. Complementary transcriptomic profiling identified significant dysregulation of oxidative phosphorylation components, providing mechanistic insights into PIC’s dual-mode action on parasite bioenergetics and organellar integrity.

## 2. Materials and Methods

### 2.1. Ethics Approval and Consent to Participate

All murine procedures were performed in compliance with the Guide for the Care and Use of Laboratory Animals of the Ministry of Science and Technology of China. The animal studies were reviewed and approved by the Institutional Animal Care and Use Committee of Guangxi University (approval number: Gxu-2022-078).

### 2.2. Culture of Cells and Parasites

Human foreskin fibroblasts (HFFs) and Vero cells (African green monkey kidney cells) were acquired from the American Type Culture Collection (ATCC, Manassas, VA, USA). Cells were maintained in high-glucose Dulbecco’s Modified Eagle Medium (DMEM) (Macgene, Beijing, China) supplemented with 10% fetal bovine serum (FBS, Gibco, Waltham, MA, USA), 100 IU/mL penicillin, and 0.1 mg/mL streptomycin (Beyotime, Shanghai, China). The Type I RH strain of *T. gondii* (TgRH-Luc), stably expressing firefly luciferase under the UPRT locus, was propagated in Vero cells. Endogenous 3×FLAG-tagged strains of F1β (TGME49_261950), ASAP16 (TGME49_201800), TOM40 (TGME49_218280), and ASAP7 (TGME49_218280) in the RH ΔKu80 background were generated as previously described [23]. Tachyzoites were cultured in 2% FBS-DMEM at 37 °C under 5% CO_2_. Piceatannol (PIC, Aladdin, Shanghai, China) was dissolved in dimethyl sulfoxide (DMSO) as a 100 mM stock.

### 2.3. Immunofluorescence Assay

Freshly harvested *T. gondii* tachyzoites were inoculated onto HFF monolayers in 12-well plates and incubated for 3 h at 37 °C/5% CO_2_. Infected cells were fixed with 4% paraformaldehyde (1 h), permeabilized with 0.25% Triton X-100 (30 min), and blocked with 3% bovine serum albumin (BSA) in PBS (30 min). Primary antibodies included rabbit anti-TgGAP45 (1:300), mouse anti-FLAG (1:50; Sigma, St. Louis, MO, USA), and rabbit anti-TgIMC1 (1:300; kindly provided by Prof. Qun Liu, China Agricultural University). After 1 h of incubation, cells were washed thrice with PBS and incubated with FITC- or Cy3-conjugated secondary antibodies (1 h). Nuclei were counterstained with Hoechst 33258 (Sigma, St. Louis, MO, USA). Fluorescent images were acquired using a Zeiss Axio Observer microscope (Zeiss, Oberkochen, Germany).

### 2.4. Detection of Mitochondrial Membrane Potential (ΔΨm)

Changes in the mitochondrial membrane potential of extracellular tachyzoites treated with PIC were evaluated using the JC-10 assay kit (Biosharp, Shanghai, China) according to the instructions of manufacturer. Briefly, fresh tachyzoites (2 × 10^6^) were treated with different concentrations of PIC (0, 30, 60, and 100 μM) for 4 h at 37 °C. Subsequently, tachyzoites were incubated in 0.5 mL of staining solution containing the potentiometric probe JC-10 for 20 min at 37 °C in the dark. The samples were then rinsed twice with buffer and resuspended in JC-10 staining buffer. Finally, the FI signals of JC-10 (JC10 monomer wavelength of Ex = 490 nm/Em = 530 nm; JC-10 polymer wavelength of Ex = 525 nm/Em = 590 nm) were detected using a fluorescence microplate reader (Tecan, Infinite M200 PRO, Männedorf, Switzerland).

The ratio of fluorescence values of J-aggregates to those of monomers was calculated using the following formula: RLU_J-aggregates_/RLU_J-Monomers_. Images were also taken for each treatment dose and control using a Zeiss Fluorescence Microscopy system (Zeiss, Oberkochen, Germany). Carbonyl cyanide 3-chlorophenylhydrazone (CCCP), a protonophore that leads to ΔΨm depletion, served as a positive control.

### 2.5. ATP Level Determination

Tachyzoites (2 × 10^6^ per experimental group) were harvested and subsequently exposed to varying concentrations of PIC (0, 25, 50, and 100 μM) for a duration of 4 h. Parasites were rapidly lysed using 200 µL of ice-cold lysis buffer. The lysed samples were then centrifuged at 12,000× *g* for 5 min. A 20 μL aliquot of the supernatant was transferred to each well of a 96-well microtiter plate, followed by the addition of 100 μL of ATP assay working solution (Beyotime, Shanghai, China). Luminescence measurements were conducted using a multilabel fluorescent microplate reader (Tecan, Infinite M200 PRO, Männedorf, Switzerland). A standard ATP curve was generated by the serial dilution of the ATP standards provided in the assay kit, which allowed for the quantification of ATP concentrations in the samples. Each experiment was conducted in triplicate.

### 2.6. ROS Assay

Vero cells infected with tachyzoites were treated with various concentrations of PIC (0, 25, 50, and 100 μM) for 2 h. For comparative analysis, tachyzoites were also treated with hydrogen peroxide (H_2_O_2_, 200 μM) to serve as a positive control, while DMSO was used as a negative control. Subsequently, fresh tachyzoites (1 × 10^6^ per group) were extracted and incubated with the 10 µM H2DCFDA (DCFHDA, 2′,7′-Dichlorodihydrofluorescein diacetate) probe for 25 min at 37 °C. Then, the parasites were washed three times with serum-free DMEM to remove excess probe. The fluorescence intensity, indicative of reactive oxygen species (ROS) production, was measured at an excitation wavelength of 488 nm and an emission wavelength of 525 nm using a fluorescence microtiter plate reader (Tecan, Infinite M200 PRO, Männedorf, Switzerland) [24]. The final ROS values for each experimental group were expressed as relative light units per parasite (RLU/parasite).

### 2.7. RNA Extraction and RNA-Seq

Vero cells infected with tachyzoites (3 × 10^7^) were treated with 100 μM PIC or DMSO for 12 h post-infection. After 2 h of incubation, uninfected tachyzoites were removed by extensive washing. Subsequently, the infected cells were treated with 100 μM PIC or DMSO for 12 h, respectively. The treated parasites were then collected, and total RNA was extracted using TRIzol reagent (Invitrogen, Carlsbad, CA, USA). The purity, concentration, and integrity of total RNA were assessed using a nanophotometer (IMPLEN, Los Angeles, CA, USA), a Qubit^®^ RNA Assay Kit (Qubit^®^ 2.0 Fluorometer) (Life Technologies, Carlsbad, CA, USA), and an RNA Nano 6000 Assay Kit (Agilent Technologies, Santa Clara, CA, USA) on a Bioanalyzer 2100 system. Only RNA samples of sufficient quality were selected for library preparation. Illumina sequencing libraries were constructed using the NEBNext^®^ Ultra™ RNA Library Prep Kit for Illumina^®^ (NEB, Ipswich, MA, USA), following the manufacturer’s protocol. Sequencing was conducted on the Illumina NovaSeq 6000 platform, generating 150 bp paired-end reads. Three biological replicates were employed for each group. The raw sequencing data were deposited in the Sequence Read Archive (SRA) database under the accession number PRJNA1252425.

### 2.8. Bioinformatics

RNA sequencing data analysis was conducted on the BMKCloud platform (Accessed on 17 August 2023: https://www.biocloud.net/). Paired-end high-quality reads were aligned to the *T. gondii* ME49 reference genome (ToxoDB release 57) using TopHat2 v2.1.1 [25] with default parameters. Transcript assembly and quantification were performed using Cufflinks v2.2.1 [26]. Differential gene expression analysis between PIC-treated and DMSO-treated tachyzoites was executed through the DESeq2 package in R software (version 3.6.3) [27], employing a negative binomial generalized linear model. Significantly differentially expressed genes (DEGs) were defined as those exhibiting |log2 (fold change)| > 1 with an adjusted *p*-value < 0.05 (Benjamini–Hochberg correction). Functional annotation of DEGs was performed via Gene Ontology (GO) and Kyoto Encyclopedia of Genes and Genomes (KEGG) pathway enrichment analyses using topGO (v2.54.0) [28], with the statistical significance threshold set at FDR < 0.05.

### 2.9. In Vivo Anti-Toxoplasma Efficacy Evaluation

Twenty-five female BALB/c mice (6-week-old) were randomly divided into five groups (*n* = 5/group) for anti-*Toxoplasma* assessment. An acute infection model was established via intraperitoneal injection of 100 ME49-strain tachyzoites (Groups 1–3) or 1000 Pru-strain tachyzoites (Groups 4–5), respectively. Pharmacological interventions initiated 4 h post-infection included the following: Group 1 (acute model control) received 0.1% DMSO in saline, Group 2 (acute treatment) was administered piceatannol (50 mg/kg/day), Group 3 (acute positive control) was treated with sulfadiazine (50 mg/kg/day) via oral gavage, Group 4 (Pru infection model control) received 0.1% DMSO in saline, and Group 5 (Pru infection treatment) was administered piceatannol (50 mg/kg/day). Survival parameters (mortality events, time to death) and clinical manifestations (weight loss, neurological signs) were recorded daily. Kaplan–Meier survival curves were generated to evaluate therapeutic efficacy across experimental groups.

### 2.10. Statistical Analyses

Statistical significance in the plaque assay, invasion, proliferation, and parasite growth inhibition assays was evaluated by two-tailed unpaired *t*-tests, while the significance of survival was determined by the log-rank Mantel–Cox test using GraphPad Prism 9 (San Diego, CA, USA). Statistical data are expressed as the mean value ± standard deviation of data from at least three independent experiments.

## 3. Results

### 3.1. Influence of PIC on Mitochondrial Morphology in T. gondii Tachyzoites

**Figure 1:**
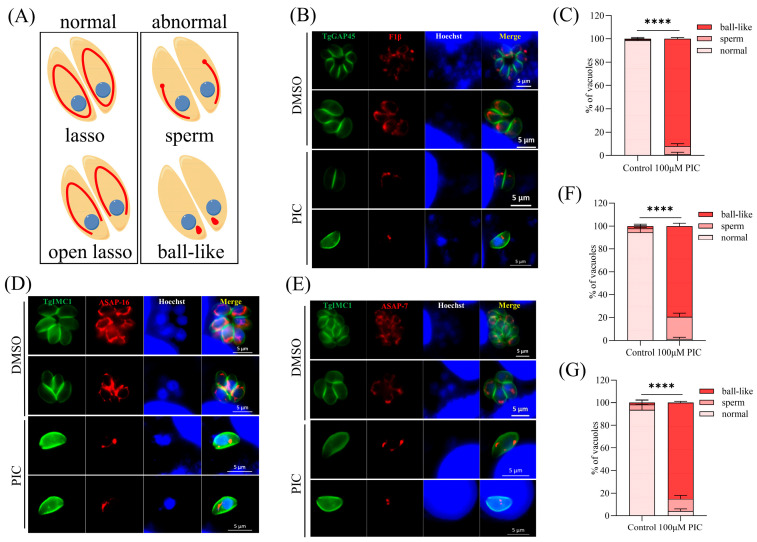
Mitochondrial inner membrane defects induced by PIC treatment: (**A**) Schematic representation of the distinct mitochondrial morphologies observed in *T. gondii*. Intracellular parasite mitochondria were visualized by immunofluorescence assay (IFA) using FLAG-tagged F1β. (**B**) IFA of GAP45 and F1β. Tachyzoites (3 × 10^5^) were allowed to invade HFF monolayers on coverslips and then treated with DMSO or 100 μM PIC for 12 h. Parasite outlines were labeled with rabbit anti-GAP45 (green), and nuclei were stained with Hoechst 33342 (blue). Scale bar = 5 μm. (**C**) Quantification of mitochondrial morphology classes in 100 randomly selected tachyzoites from three independent experiments. Statistical analysis was performed using Student’s *t*-test (**** *p* < 0.0001). (**D**,**E**) ASAP16 (**D**) and ASAP7 (**E**) parasites were treated with DMSO or 100 μM PIC for 12 h and then subjected to IFA using mouse anti-FLAG and rabbit anti-TgIMC1 antibodies. Parasite shape was visualized with anti-TgIMC1 (green) and nuclear DNA stained with Hoechst 33342 (blue). Scale bar = 5 μm. (**F**,**G**) Quantification of mitochondrial morphology in ASAP16-FLAG (**F**) and ASAP7-FLAG (**G**) strains after 12 h of PIC treatment. Data represent counts of 100 randomly selected tachyzoites per strain from three independent experiments. Statistical significance was determined by Student’s *t*-test (**** *p* < 0.0001).

Given prior evidence implicating PIC in targeting the α, β, and γ subunits of mitochondrial ATP synthase in *E. coli* [22], we investigated its effects on *T. gondii* bioenergetics using CRISPR/Cas9-engineered RH ΔKu80 parasites endogenously expressing a C-terminal 3 × FLAG-tagged F1β subunit (ATP5B; TGME49_261950) [23]. Intracellular tachyzoites typically exhibit perinuclear “lasso”-patterned mitochondrial networks, whereas extracellular parasites display condensed “sperm-like” or globular (“ball-shaped”) mitochondrial conformations [29] (Figure 1A). Immunofluorescence analysis revealed that F1β typically encircled the nucleus in parasites treated with DMSO. However, following PIC treatment, 99% of the parasites exhibited abnormal F1β localization (Figure 1B,C). Given that the mitochondrial ATP synthase complex subunit F1β is typically localized on the inner mitochondrial membrane, the altered F1β localization post-PIC treatment piqued our interest. To ascertain whether F1β mislocalization was a direct effect of PIC on the protein itself or a consequence of perturbed mitochondrial inner membrane morphology, we examined the localization of two additional F_o_ subunits of the *T. gondii* ATP synthase complex: ASAP16 (TGME49_201800) and ASAP7 (TGME49_21894) [30]. Parasites expressing C-terminal FLAG-tagged TgASAP16 and TgASAP7 were treated with PIC. Compared to the untreated controls, the localization of ASAP16 and ASAP7 in PIC-treated tachyzoites was also abnormal (Figure 1D,E). These proteins failed to surround the nucleus in the expected lasso pattern, instead aggregating into irregular configurations, with a significant difference noted relative to the DMSO group (Figure 1F,G).

**Figure 2:**
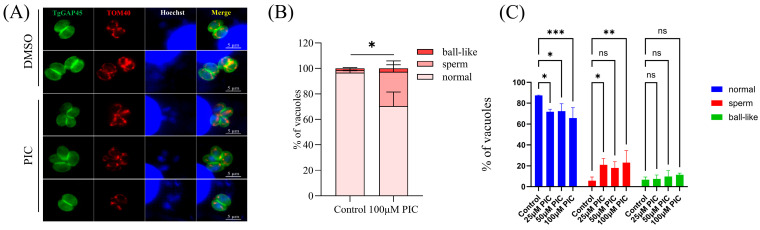
Morphological alterations in the parasite mitochondrial outer membrane following PIC treatment: (**A**) Immunofluorescence analysis of the *T. gondii* outer mitochondrial membrane. Host-cell-cultured TgTOM40–FLAG parasites were treated with 100 µM PIC or DMSO vehicle, and TOM40 localization was detected by IFA using an anti-FLAG antibody (red). Parasite boundaries were labeled with anti-GAP45 (green), and nuclei were stained with Hoechst dye (blue). Scale bar = 5 µm. (**B**) Quantification of outer mitochondrial membrane morphology in 100 randomly selected tachyzoites per condition from three independent experiments. Statistical significance was determined by Student’s *t*-test (* *p* < 0.05). (**C**) Immunofluorescence staining of *T. gondii* mitochondria with MitoTracker. Tachyzoites were incubated with graded concentrations of PIC (0–100 µM) for 12 h and then stained with MitoTracker Red CMXRos (Beyotime, Shanghai, China). Mitochondrial morphologies were quantified in 100 randomly selected parasites per experiment by scoring anti-FLAG-labeled TOM40. Statistical analysis was performed using two-way ANOVA with Tukey’s post hoc test (ns—not significant; * *p* < 0.05; ** *p* < 0.01; *** *p* < 0.001).

To confirm whether PIC can affect *T. gondii* mitochondrial outer membrane morphology, the endogenous FLAG label was added to the C-terminal of mitochondrial outer membrane marker TOM40 [23]. Quantitative image analysis revealed PIC-induced structural anomalies in 29.67% of intracellular tachyzoites, comprising 2.67% ball-shaped and 27% sperm-shaped mitochondrial conformations (Figure 2A,B), which is significantly elevated compared to the DMSO control.

In addition, we further studied the mitochondrial morphology of extracellular tachyzoites treated with PIC by IFA using MitoTracker staining (Beyotime, Shanghai, China). A previous study showed that extracellular *Toxoplasma* tachyzoites also have three mitochondrial morphologies detected by immunofluorescence microscopy [31]. Our results indicated a significant increase in sperm-like mitochondria and a significant decrease in normal mitochondrial morphology in extracellular tachyzoites treated with PIC (Figure 2C).

### 3.2. PIC Modulates Oxidative Phosphorylation Gene Transcription in T. gondii

**Figure 3:**
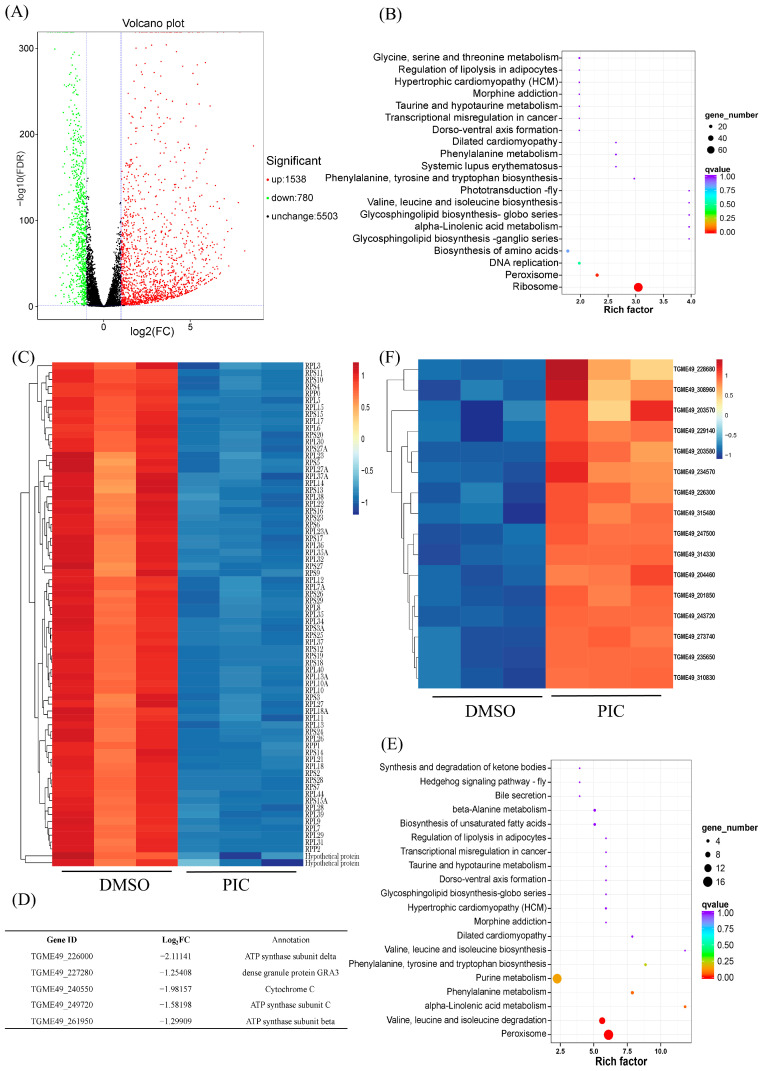
PIC-induced alterations in mitochondrial gene expression in *T. gondii*: (**A**) Volcano plot showing global gene expression changes in *T. gondii* after PIC treatment. Significantly upregulated and downregulated genes are highlighted in red and green, respectively. (**B**) KEGG pathway enrichment analysis of downregulated DEGs. The X-axis represents the rich factor, the Y-axis lists the KEGG pathways, the dot size indicates the number of DEGs, and the dot color corresponds to adjusted *p*-values. (**C**) Heatmap illustrating downregulation of ribosomal genes before and after PIC treatment; gene names are indicated to the right (Log2FC < −1; *p* < 0.01). (**D**) Expression profiles of five DEGs related to the oxidative phosphorylation pathway. (**E**) KEGG pathway enrichment analysis of upregulated DEGs. (**F**) Heatmap showing upregulation of peroxisome genes before and after PIC treatment; gene IDs are indicated to the right (Log2FC > 1; *p* < 0.01).

To elucidate the mechanism by which PIC induces mitochondrial injury in *T. gondii*, we performed RNA-seq on parasites treated with 100 μM PIC for 12 h. Differential expression analysis with DESeq2 identified 2318 DEGs (|log_2_FC| > 1, FDR < 0.05), of which 1538 were upregulated, 780 downregulated, and 5503 unchanged (Figure 3A, Supplementary Data S1).

KEGG pathway annotation revealed that PIC likely impairs parasite growth by suppressing the ribosome, peroxisome, and DNA replication pathways. An analysis of downregulated DEGs showed significant enrichment in ribosomal genes, with 73 ribosome-associated transcripts markedly decreased (Figure 3B,C), which may underlie the growth-inhibitory effect of PIC. Additionally, genes involved in DNA replication were downregulated following PIC treatment (Figure 3B), potentially contributing to the observed defects in tachyzoite division.

Given our morphological observations of inner mitochondrial membrane disruption, we next examined the impact of PIC on transcripts encoding oxidative phosphorylation components. PIC treatment elicited significant alterations in the transcription of genes for respiratory chain complexes III and IV, as well as complex V (ATP synthase), with the β-, δ-, and c-subunit-encoding genes of ATP synthase significantly downregulated (Figure 3D and Figure S1). Such transcriptional changes likely account for the aberrant mitochondrial inner membrane structure. Conversely, an analysis of upregulated DEGs revealed that transcripts of peroxisome-associated genes were significantly elevated after PIC exposure (Figure 3E,F), suggesting a compensatory peroxisomal response to mitochondrial dysfunction.

### 3.3. Mitochondrial Dysfunction Induced by PIC

**Figure 4:**
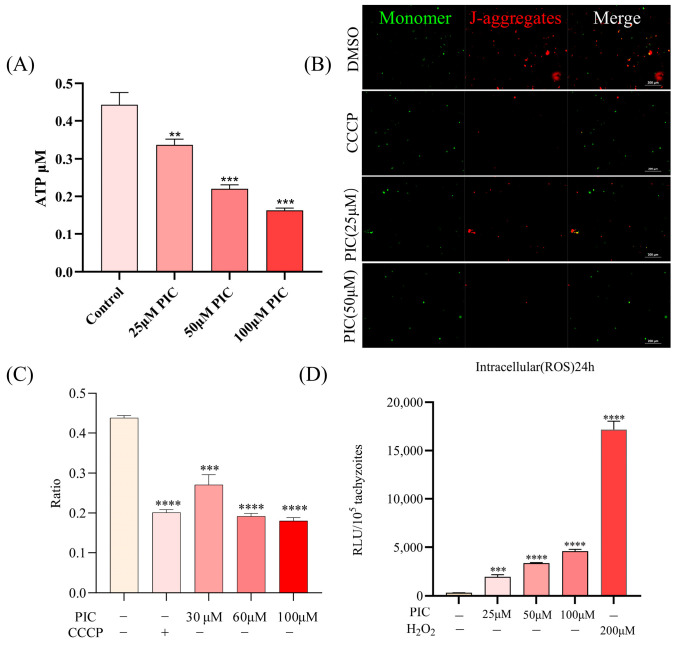
Mitochondrial dysfunction induced by PIC treatment: (**A**) Dose-dependent reduction in intracellular ATP in *T. gondii*. Extracellular tachyzoites (2 × 10^6^ per sample) were incubated with 0, 25, 50, or 100 μM PIC for 4 h at 37 °C. Parasites were lysed, and ATP levels were quantified using a multimode plate reader. (**B**,**C**) Assessment of mitochondrial membrane potential (ΔΨm) in PIC-treated tachyzoites using the JC-10 probe. J-Monomers (red) and J-aggregates (green) represent parasites with low and high mitochondrial membrane potentials, respectively. The ratio of aggregate to monomer fluorescence (RLU_aggregates/RLU_monomers) is presented for each treatment group. (**D**) Measurement of reactive oxygen species (ROS) in tachyzoites (1 × 10^5^) following 24 h PIC exposure, using the DCFH-DA probe. Data are mean ± SD; statistical significance is denoted as ** *p* < 0.01, *** *p* < 0.001, and **** *p* < 0.0001.

Since PIC affected the transcriptional level and localization of the parasite mitochondrial ATP synthase complex subunit, we assessed whether PIC impairs mitochondrial function. First, we measured intracellular ATP in tachyzoites following PIC treatment. Our results showed a significant, dose-dependent decrease in ATP levels following PIC treatment (Figure 4A). ATP synthesis is mainly driven by proton movement, with ΔΨm being crucial for this process, established by proton influx from the matrix into the intermembrane space. Given the marked reduction in ATP levels after PIC exposure, we hypothesized that ΔΨm would be compromised by PIC. Using the ΔΨm-sensitive fluorescent probe JC-10, with CCCP as a positive control, we detected a progressive decrease in the red/green fluorescence ratio with an increasing PIC concentration (Figure 4B,C), indicating a clear dose-dependent loss of ΔΨm. These findings suggest that PIC reduces the mitochondrial membrane potential of *T. gondii* in a dose-dependent manner.

Mitochondrial impairment in *T. gondii* is known to promote intracellular reactive oxygen species (ROS) accumulation [32,33]. Accordingly, we quantified ROS in tachyzoites treated with escalating doses of PIC for 24 h and found a significant, concentration-dependent increase in ROS levels (Figure 4D). These data aligned with the upregulation of peroxisome-related transcripts (Figure 3F, reflecting an oxidative stress response.

### 3.4. Treatment with PIC Reduces Parasite Virulence in Mice

**Figure 5:**
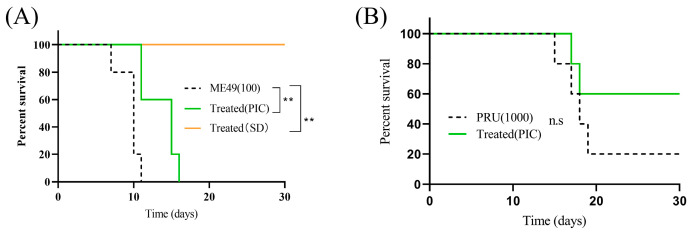
Treatment with PIC reduces parasite virulence in mice: (**A**) Six-week-old female BALB/c mice (*n* = 5) were infected intraperitoneally with 100 freshly isolated ME49 tachyzoites. Experimental group was treated with PIC (50 mg/kg), positive control group was administered sulfadiazine (SD) (50 mg/kg), and negative control group was given DMSO + normal saline. (**B**) Six-week-old female BALB/c mice (*n* = 5) were infected intraperitoneally with 1000 Pru tachyzoites and administered PIC (50 mg/kg). ns—not significant; ** *p* < 0.01.

Our previous work demonstrated that PIC delays mortality in mice acutely infected with the RH strain [21]. Here, we evaluated the efficacy of oral PIC against Type II ME49 and Pru strains. In ME49-infected mice, PIC administration deferred the onset of death to day 11 (40% mortality), with an additional 40% succumbing on day 15 and complete mortality by day 16. In contrast, DMSO-treated controls began to die on day 7 (20% mortality), with mortality reaching 80% by day 10, and were all deceased by day 11 (Figure 5A). In Pru-infected mice, PIC treatment resulted in single deaths on days 17 and 18, with three survivors through day 30; DMSO controls exhibited initial mortality on day 15 and one death each on days 17–19 (Figure 5B). These findings indicate that PIC significantly prolongs host survival but does not achieve complete parasite clearance.

## 4. Discussion

There is growing interest in natural plant extracts as novel anti-*T. gondii* agents. Natural compounds often combine high bioactivity and efficacy with low toxicity and cost. For example, artemisinin and its derivatives are renowned for their potent antimalarial properties and have been actively investigated against *T. gondii* in vivo [34,35]. Resveratrol, a natural polyphenol, reduces extracellular tachyzoite proliferation by disrupting the parasite’s redox homeostasis and alleviating cellular stress to promote host-cell apoptosis [36].

PIC, a natural analog of resveratrol found in passion fruit, blueberries, grapes, sugarcane, white tea, and rhubarb, possesses diverse bioactivities—including antioxidant, antiproliferative, immunomodulatory, anti-inflammatory, antithrombotic, anticancer, lipid-lowering, and antimicrobial effects [19,37]. In our previous study, PIC demonstrated potent anti-*T. gondii* activity: it inhibited both intracellular and extracellular RH tachyzoites with an EC_50_ of 28.10 μM [21].

Prior investigations suggested that PIC may inhibit bacterial ATP synthase by interacting with the active pocket formed by its α and β subunits and the γ-subunit C-terminal region [22]. We first examined the localization of the ATP synthase F1β subunit (an inner membrane marker) and observed aberrant mitochondrial distribution following PIC treatment. Subsequent analysis of two additional subunits, ASAP16 and ASAP7, revealed similar mislocalization: instead of the canonical perinuclear ring, these proteins aggregated into irregular foci. Given the mislocalization of multiple inner membrane proteins, we hypothesized that PIC induces global mitochondrial abnormalities. An assessment of the outer membrane marker TOM40 confirmed abnormal localization, albeit at a significantly lower incidence than inner membrane markers. Together, these findings indicate that PIC primarily disrupts the tachyzoite inner mitochondrial membrane, with secondary effects on outer membrane integrity.

Given the pronounced mitochondrial morphological defects induced by PIC, we investigated its impact on mitochondrial function. Building on the known ability of resveratrol to disrupt parasite redox balance and induce apoptosis [36], we performed RNA-seq on PIC-treated tachyzoites. Transcriptomic analysis revealed that PIC significantly affects pathways related to ribosomal proteins, peroxidases, mitochondrial respiratory chain complexes, and DNA replication. Notably, genes encoding ribosomal components and DNA replication factors were markedly downregulated, likely contributing to PIC’s antiproliferative effect. Furthermore, transcripts encoding multiple respiratory chain proteins—particularly those of complexes III, IV, and V (including the β, δ, and c subunits of ATP synthase)—were significantly repressed. As these proteins reside in the inner mitochondrial membrane and are essential for ATP synthesis, their transcriptional downregulation correlates with observed mitochondrial inner membrane disruption and reduced ATP levels.

Reactive oxygen species (ROS), byproducts of aerobic metabolism, play critical roles in cellular physiology [38]. Mitochondria are the principal ROS source via the electron transport chain, with complex III being a major contributor [39,40,41]. Following PIC treatment, we observed the upregulation of complex III transcripts alongside elevated ROS levels, suggesting that enhanced ROS production is linked to transcriptional changes in oxidative phosphorylation components. Moreover, mitochondrial ΔΨm is a key functional indicator; PIC-treated tachyzoites exhibited concomitant ROS elevation and ΔΨm collapse, indicating that PIC-induced excessive ROS disrupts membrane potential and impairs ATP synthesis.

Despite its potent in vitro anti-*T. gondii* activity and lack of host cytotoxicity, PIC’s efficacy in vivo is limited, which is likely related to its pharmacokinetics in mice. In Pru-strain-infected mice, oral PIC reduced mortality but did not prevent eventual death, and in ME49-strain infections, PIC treatment only extended survival without achieving clearance. Notably, the therapeutic effects of PIC showed no obvious dose dependency. In our previous study, mice treated with a relatively low dose of 15 mg/kg/day of PIC all died by day 15 [21]. In the present study, even when the PIC dose was increased to 50 mg/kg/day, all mice still died by day 16. Thus, the higher dose of PIC only resulted in a one-day postponement of death. Rapid in vivo clearance of PIC likely underlies these outcomes. The peak plasma levels in rats occur within 15 min of oral dosing, decline markedly by 2 h, and are undetectable by 4 h, with extensive urinary metabolites [42]. As a consequence, the plasma concentration of PIC in mice cannot be sustained within the therapeutic range for a sufficiently long time to achieve effective parasite clearance. Even though increasing the dose can prolong the time taken for metabolism to some extent, it cannot fundamentally solve the problem of PIC’s short half-life. Therefore, the structural optimization of PIC to prolong its half-life may enhance its therapeutic efficacy against *T. gondii* in vivo.

## Data Availability

All raw sequences have been deposited in the NCBI Sequence Read Archive with the accession number PRJNA1252425 (https://www.ncbi.nlm.nih.gov/bioproject/PRJNA1252425, accessed on 18 April 2025).

## Supplementary Materials

The following supporting information can be downloaded at https://www.mdpi.com/article/10.3390/microorganisms13061203/s1, Supplementary Data S1: Differentially expressed *T. gondii* genes after PIC treatment. *T. gondii* tachyzoites of RH in HFF cells were treated with 100 μM PIC for 12 h, and then the differentially expressed genes were analyzed by RNA-seq; Figure S1: KEGG map of the oxidative phosphorylation pathway of *T. gondii* tachyzoites before and after PIC treatment.
