# Supplementary figures and images for: Piceatannol Induces Mitochondrial Dysfunction in Toxoplasma gondii
Source: Microorganisms. 2025 May 25;13(6):1203. doi: 10.3390/microorganisms13061203 (PMC12194955; doi:10.3390/microorganisms13061203)

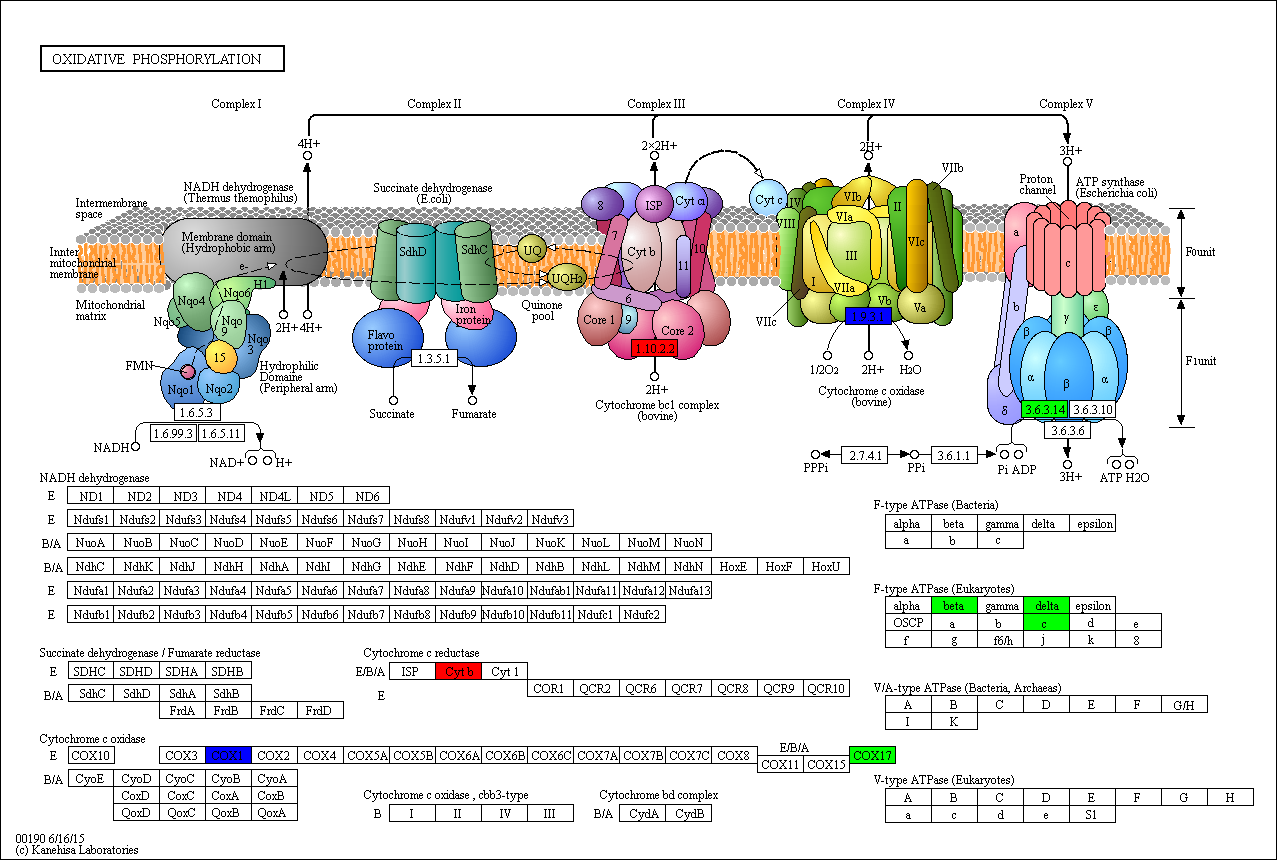

Supplement: Supplementary file 1 [file microorganisms-13-01203-s001.zip › Figure S1.tif]
